# Structural Analysis of the Interaction between the Bacterial Cell Division Proteins FtsQ and FtsB

**DOI:** 10.1128/mBio.01346-18

**Published:** 2018-09-11

**Authors:** Danguole Kureisaite-Ciziene, Aravindan Varadajan, Stephen H. McLaughlin, Marjolein Glas, Alejandro Montón Silva, Rosa Luirink, Carolin Mueller, Tanneke den Blaauwen, Tom N. Grossmann, Joen Luirink, Jan Löwe

**Affiliations:** a MRC Laboratory of Molecular Biology, Cambridge, United Kingdom; b Amsterdam Institute of Molecules, Medicines and Systems, VU University, Amsterdam, The Netherlands; c Bacterial Cell Biology and Physiology, Swammerdam Institute for Life Sciences, University of Amsterdam, Amsterdam, The Netherlands; University of Kansas Medical Center; Harvard University

**Keywords:** FtsL, FtsN, X-ray crystallography, bacterial cell division, biochemistry, divisome, molecular microbiology, periplasm, protein structure-function

## Abstract

Most bacteria and archaea use the tubulin homologue FtsZ as its central organizer of cell division. In Gram-negative Escherichia coli bacteria, FtsZ recruits cytosolic, transmembrane, periplasmic, and outer membrane proteins, assembling the divisome that facilitates bacterial cell division. One such divisome component, FtsQ, a bitopic membrane protein with a globular domain in the periplasm, has been shown to interact with many other divisome proteins. Despite its otherwise unknown function, it has been shown to be a major divisome interaction hub. Here, we investigated the interactions of FtsQ with FtsB and FtsL, two small bitopic membrane proteins that act immediately downstream of FtsQ. We show in biochemical assays that the periplasmic domains of E. coli FtsB and FtsL interact with FtsQ, but not with each other. Our crystal structure of FtsB bound to the β domain of FtsQ shows that only residues 64 to 87 of FtsB interact with FtsQ. A synthetic peptide comprising those 24 FtsB residues recapitulates the FtsQ-FtsB interactions. Protein deletions and structure-guided mutant analyses validate the structure. Furthermore, the same structure-guided mutants show cell division defects *in vivo* that are consistent with our structure of the FtsQ-FtsB complex that shows their interactions as they occur during cell division. Our work provides intricate details of the interactions within the divisome and also provides a tantalizing view of a highly conserved protein interaction in the periplasm of bacteria that is an excellent target for cell division inhibitor searches.

## Abstract (precis)

In most bacteria and archaea, filaments of FtsZ protein organize cell division. FtsZ forms a ring structure at the division site and starts the recruitment of 10 to 20 downstream proteins that together form a multiprotein complex termed the divisome. The divisome is thought to facilitate many of the steps required to make two cells out of one. FtsQ and FtsB are part of the divisome, with FtsQ being a central hub, interacting with most of the other divisome components. Here we show for the first time in detail how FtsQ interacts with its downstream partner FtsB and show that mutations that disturb the interface between the two proteins effectively inhibit cell division.

## INTRODUCTION

The divisome is a macromolecular complex formed by at least 12 essential proteins and even more nonessential proteins. It facilitates bacterial cell division through a number of processes, including cell constriction, synthesis of the septal peptidoglycan (PG) wall, and ultimately, cell separation (1–5). In Escherichia coli, divisome assembly starts with formation of a ring structure localized midcell, containing the bacterial tubulin homologue FtsZ in the cytoplasm (6, 7) and anchoring of the ring in the inner membrane by FtsA and ZipA (8, 9). This is followed by recruitment of additional cell division proteins FtsEX, FtsK, FtsQ, FtsL, FtsB, FtsW, FtsI, and FtsN in order of their localization interdependence, all of them transmembrane proteins. The functions of several of these divisome proteins have been deduced, such as the role of FtsEX in transmembrane regulation of septal PG hydrolytic enzymes (10), the role of FtsK in XerCD-mediated chromosome decatenation (11), and the proposed role of FtsW/FtsI(PBP3) as a hybrid septal peptidoglycan synthase with transglycosylase and transpeptidase activities, respectively (12). FtsN has a particularly interesting role as the trigger for septal peptidoglycan synthesis, depending on the assembly of the entire complex, somehow affecting FtsA on the cytoplasmic side of the cell membrane directly as a feedback or even checkpoint mechanism (13). FtsQLB, FtsI, and FtsN have only single transmembrane helices anchoring them in the membrane (bitopic membrane proteins) in addition to globular periplasmic domains, where they act on/react to PG synthesis.

FtsQ is considered to play a central, yet enigmatic role in assembly of the divisome through a multitude of interactions, as no enzymatic function is known for this protein (14). Two-hybrid analyses have suggested that FtsQ interacts with ~10 cell division proteins of which the interactions with FtsB and FtsL were confirmed biochemically (15). The FtsQBL complex may form independently before its recruitment to midcell by FtsK, where it interacts with the later divisome proteins needed for cell division (16).

FtsQ is a particularly attractive target for the development of inhibitors of protein-protein interactions (PPIs) that block bacterial division for the following reasons. (i) It is an essential protein of low abundance (~50 to 250 copies per cell) (17). (ii) It has multiple interactions in the much more easily accessible periplasm (14, 16). (iii) It has no obvious eukaryotic homologues (18). Previously, the structure of the periplasmic domain of FtsQ from E. coli has been solved by X-ray crystallography (19) and shown to consist of two subdomains, named α and β. Together with the transmembrane domain (TMD) (a single bitopic helix in FtsQ), the α domain is believed to be required for recruitment by FtsK although other interactions have been ascribed to this domain as well (19, 20). The α domain is located directly downstream from the TMD in the sequence of FtsQ and corresponds to the more broadly distributed polypeptide transport-associated (POTRA) domains that have been implicated in transient PPIs in a range of transporter proteins (21). The β domain has been implicated in multiple interactions, including those with FtsB and FtsL (19).

FtsB and FtsL are small bitopic inner membrane proteins (103 and 121 residues in E. coli, respectively). FtsB and FtsL have also been suggested to form a distinct subcomplex prior to localization to the septum, making a strictly sequential recruitment less likely. Both proteins may contain a leucine zipper motif in their periplasmic domain that together with the TMDs have prompted suggestions that FtsB and FtsL form a heterodimeric or tetrameric complex (22, 23). Recently, an *in vivo* scanning photo-cross-linking approach to map interactions of FtsQ with FtsB and FtsL at the amino acid level has been conducted, considering one-fifth of all surface-exposed residues (24). Two hot spots for the interactions with FtsBL were identified on FtsQ: one in the α domain close to the membrane around residue FtsQ R75, primarily interacting with FtsL, and a more pronounced hot spot in the conserved membrane-distal part of the β domain around residue FtsQ Y248, primarily interacting with FtsB. In addition, it was previously shown with purified proteins *in vitro* that artificially dimerized FtsB and FtsL in which their TMDs were replaced with a heterodimeric coiled-coil fragment, bind to the periplasmic portion of FtsQ (25). In those experiments, the periplasmic domain of E. coli FtsQ was copurified with the heterodimerized E. coli FtsB and FtsL constructs as a stable trimeric complex. FtsB was also shown to interact with FtsQ in the absence of FtsL, whereas FtsL on its own failed to copurify with FtsQ.

Here we present further quantitative biochemical investigations of the periplasmic interaction between FtsQ, FtsB, FtsL, and FtsN, showing that the FtsQB complex is formed with a submicromolar dissociation constant as determined by surface plasmon resonance (SPR). SPR does not indicate interactions between FtsB and FtsL but shows a trimeric FtsQLB complex. FtsN interacts independently of FtsB with FtsQ, whereas the interaction of FtsQ with FtsL is not additive with FtsB. In line with these findings, we present a crystal structure of the periplasmic domain of E. coli FtsQ in complex with the periplasmic domain of E. coli FtsB. The structure resolves residues 64 to 87 of FtsB that form an α-helix and a β-strand, linked by a loop. The region in FtsQ that interacts with FtsB has Y248 at its center and is at the membrane-distal end of the protein, which is in agreement with the previous cross-link data (24). Mutational analysis coupled with cellular microscopy and also SPR confirmed residues in the interacting region highlighted by the crystal structure that are critical for binding of FtsB to FtsQ, and consequently for functioning of these proteins in cell division.

## RESULTS

### The periplasmic domain of FtsB binds to FtsQ, but not FtsL.

**FIG 1:**
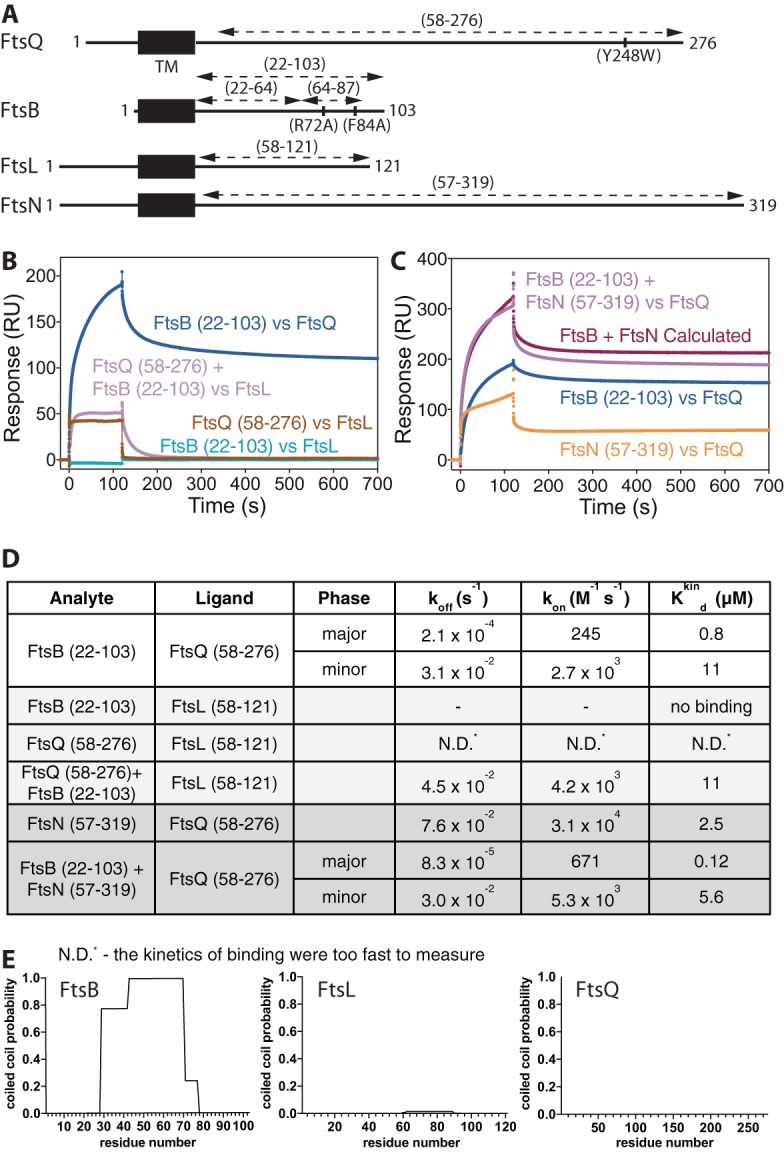
The periplasmic domains of E. coli FtsQ and FtsB form a stable complex *in vitro*. (A) Schematic drawing showing the various periplasmic constructs of FtsQ, FtsB, FtsL, and FtsN used throughout the study. TM, transmembrane domain. (B) Surface plasmon resonance (SPR) experiments investigating the interactions of the periplasmic domains of E. coli FtsQ, FtsB, and FtsL. FtsB binds to FtsQ. FtsL binds to FtsQ, and FtsL and FtsB together also bind to FtsQ, although synergistically, not independently. FtsB and FtsL do not bind to each other in isolation. Note that the proteins mentioned last were immobilized. (C) SPR investigation of the interaction of the periplasmic domain of E. coli FtsN with FtsQ. A control, showing that binding of FtsB and FtsN to FtsQ is additive, which means that they bind independently to FtsQ, different to how FtsB and FtsL bind. (D) Summary table showing quantitative analysis of the SPR data presented in panels B and C. N.D., not determined (the kinetics of binding were too fast to measure). (E) Coiled-coil predictions of full-length E. coli proteins FtsB, FtsL, and FtsQ, calculated with COILS (26). Note that only FtsB shows significant coiled-coil prediction, between residues ~29 and 70 (or 77). This makes it unlikely that FtsB and FtsL form a canonical heteromeric coiled coil, and we show in panel A that they do not interact *in vitro* on their own. Please note that also predictions with 2ZIP (27) were negative for FtsL (but positive for FtsB) (not shown).

To understand the periplasmic interactions of FtsB with FtsQ and FtsL, we performed surface plasmon resonance (SPR) experiments with purified protein domains (Fig. 1A to D). The periplasmic domain of E. coli FtsB comprising residues 22 to 103 binds strongly to E. coli FtsQ (residues 58 to 276) immobilized on the SPR chip, with a major *K*_*d*_ (dissociation constant) of 0.8 µM. The periplasmic domain of FtsL also binds to FtsQ; however, in this experiment, FtsL was immobilized, and the binding and release were so fast that parameters could not be determined reliably. Surprisingly, when FtsB was added to immobilized FtsL, no binding could be detected. To investigate this further, FtsB and FtsQ were added together to immobilized FtsL, resulting in measurable binding with a *K*_*d*_ of 11 µM. The lack of binding of FtsL alone to FtsB is surprising because it has been proposed that FtsB and FtsL form a coiled-coil/leucine zipper complex with each other and when binding to FtsQ (22, 23). When analyzing the propensity of FtsL and FtsB to form coiled coils with COILS (Fig. 1E) (26), only E. coli FtsB showed significant coiled-coil content between residues 29 and 70 (or 77). 2ZIP leucine zipper predictions (27) were also negative for FtsL but positive for FtsB (not shown). We think given these data it is unlikely that the periplasmic domains of FtsB and FtsL form a 1:1 coiled-coil/leucine zipper complex, and we could detect no binding of their periplasmic domains biochemically. The possibility that the absence of the transmembrane domains from both proteins caused this negative result cannot be excluded, since significant binding propensity could be located in the transmembrane helices of FtsB and FtsL. However, the periplasmic domains of FtsQ and FtsB together do bind to FtsL and the binding is not additive in the sense that if FtsL and FtsB each bound to FtsQ with their independent binding sites on FtsQ, the resulting binding would be stronger than their individual binding, which is not the case. To test this further, we investigated the binding of the periplasmic domain of FtsN to immobilized FtsQ as a control (Fig. 1C and D). E. coli FtsN comprising residues 57 to 319 was bound to immobilized FtsQ using SPR, and it bound with a *K*_*d*_ of 2.5 µM. When FtsB and FtsN were bound to FtsQ together, a tighter *K*_*d*_ of 0.12 µM resulted, and the resulting binding could be simulated by assuming additive binding, which would mean that FtsB and FtsN have independent, distinct binding sites on FtsQ, as shown by the calculated plot in Fig. 1C. We conclude that FtsB and FtsL alone bind well to FtsQ, and as a complex FtsB and FtsL bind to FtsQ dependent on each other, not binding to their own, independent binding sites on the surface of FtsQ. The periplasmic, soluble domains of FtsB and FtsL do not bind to each other in isolation, and only FtsB is predicted to form a coiled coil, possibly with itself.

### Crystal structure of the periplasmic FtsQ-FtsB complex.

**FIG 2:**
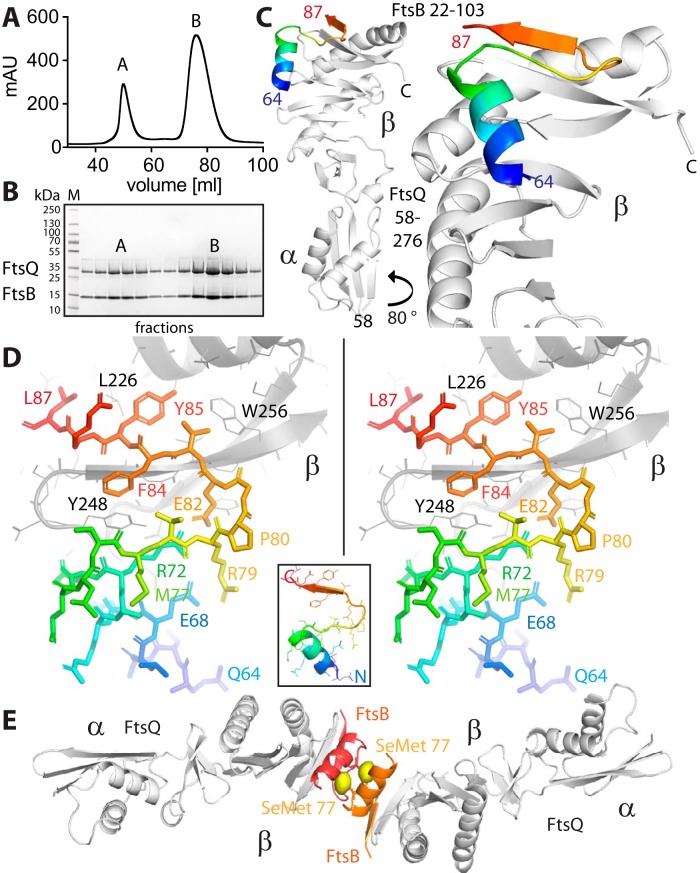
Crystal structure of the complex between the periplasmic domains of E. coli FtsB and FtsQ. (A) Size exclusion profile showing elution of the FtsQB complex. (B) SDS-PAGE of the FtsQB complex. The complex elutes as two peaks (see Fig. 5A and Fig. S6A for multiple angle light scattering [SEC-MALS] and analytic ultracentrifugation [AUC] data on the same complex) that both produced crystals and are most likely related to oligomerization or dimerization of FtsB. mAU, milli-arbitrary units. (C) Crystal structure of the complex determined to 2.6-Å resolution by molecular replacement. Crystallographic data are listed in Table 1. Only residues 64 to 87 of FtsB are resolved in the structure. FtsB forms a short helix, a connecting loop, and a β-sheet that aligns in an antiparallel orientation with the last strand of the β domain of FtsQ. (D) Stereo plot of FtsB 64–87 in stick representation, showing key residues involved in interactions with FtsQ and also internal contacts that are important for FtsB to adopt this particular structure. The structure is colored from the N terminus (blue) to the C terminus (red). The inset shows the same orientation as a ribbon plot. (E) In the crystals, FtsB forms a tight dimer that buries hydrophobic residues, including methionine 77. To be certain about the register of the amino acids of FtsB, we replaced M77 with selenomethionine (SeMet) and performed a single-wavelength anomalous diffraction (SAD) experiment (Table 1). The resulting phased anomalous difference density highlights the only methionine in FtsB in the correct location, validating our interpretation.

Inspired by the SPR results, we purified the complex between the periplasmic domains of E. coli FtsB (residues 22 to 103) and FtsQ (residues 58 to 276). The final size exclusion chromatogram of the purification is shown in Fig. 2A, showing two peaks. Both peaks yielded the same crystals, and peak A, of unexpected high apparent molecular weight is presumed to be an oligomeric state of the complex that partially dissociated on the column. Both peaks A and B elute as a defined complex with 1:1 stoichiometry (Fig. 2B). We obtained tetragonal crystals of the complex and solved the X-ray crystal structure to 2.6-Å resolution by molecular replacement with a previous E. coli FtsQ structure (Protein Data Bank [PDB] identifier [ID] 2VH1) (Table 1) (19). The resulting structure shows the previously reported two-domain architecture of FtsQ (α and β domains) largely unchanged (root mean square deviation [RMSD] between PDB ID 2VH1 chain A and the new structure is 0.85 Å over 1,298 atoms and 1.5 Å with chain B of 2VH1). FtsB binds to the C-terminal β domain of FtsQ, presumably furthest away from the membrane (Fig. 2C; also see Fig. 5B). Only a C-terminal portion of FtsB was resolved in the crystals, comprising residues 64 to 87. FtsB was not obviously degraded in the crystals where checked by SDS-PAGE, so we concluded that residues 22 to 63 and 88 to 103 were disordered. FtsB forms a helix, followed by a linker largely in β-strand conformation, leading to the final β-strand that extends the central β-sheet of the FtsQ β domain by binding to its last β-strand (Fig. 2C). Analysis of the binding mode of FtsB to FtsQ reveals a number of key interactions that are depicted in the stereographic Fig. 2D. The only helix resolved in FtsB, approximately residues 65 to 75, makes a number of charged interactions with the rest of FtsB and FtsQ, most notably two salt bridges that may be involved in stabilizing FtsB’s conformation (E68-R79 and R72-E82). Both salt bridges are also involved in binding to FtsQ via D245 and R247. Further charged or polar interactions with FtsQ are mediated by FtsB E65, E69, and N73 (not highlighted in Fig. 2D). In the crystals, the resolved part of FtsB forms a tight dimer, the significance of which we have not investigated (Fig. 2E). Figure 2E also shows the phased anomalous difference density from a single-wavelength anomalous diffraction (SAD) experiment with selenomethionine substituted M77, validating the model building, in order to produce absolute certainty for the chain trace, since the resolved FtsB domain is small.

**TABLE 1:** Crystallographic data

Parameter	Value(s) for E. coli periplasmic FtsQB complex		
	Alone	SeMet	FtsB peptide
NCBI database IDs	FtsQ: FTSQ_ECOLI	FtsQ: FTSQ_ECOLI	FtsQ: FTSQ_ECOLI
	FtsB: FTSB_ECOLI	FtsB: FTSB_ECOLI	FtsB: FTSB_ECOLI
Constructs	Q: M-58-276-GSH_6_	Q: M-58-276-GSH_6_	Q: M-58-276-GSH_6_
	B: pwd	B: MGSSHHHHHHSSGLVPRGSHM-22-103	B: 64–87 (synthetic, acetylated, M77 replaced with norleucin)
	-22-103	RGSHM-22-103	
Method	Molecular replacement	Selenium SAD	Molecular replacement

Data collection statistics			
Beamline/source	Diamond I04-1	Diamond I03	Diamond I04
Wavelength (Å)	0.92819	0.97980	0.97950

Crystal/helical			
Space/point group	P4_1_2_1_2	P4_1_2_1_2	C2
Cell (Å)	93.8, 93.8, 106.4	92.5, 92.5, 105.2	117.2, 48.0, 131.6, 112.4°

Data			
Resolution (Å)	2.6	2.8	2.8
Completeness (%)^a^	99.5 (99.5)	100.0 (99.9)	98.5 (99.7)
Multiplicity^a^	12.8 (13.4)	14.0 (13.8)	3.2 (3.3)
(*I*)/σ(*I*)^a^	22.6 (2.1)	33.3 (3.9)	10.6 (1.8)
*R*_merge_^a^	0.082 (1.702)	0.078 (1.130)	0.077 (0.744)
*R*_pim_^a^	0.024 (0.477)	0.021 (0.314)	0.051 (0.488)
CC1/2	0.999 (0.821)	1.000 (0.944)	0.998 (0.686)
Anomalous correlation		0.833 (0.010)	
Selenium sites		1	

Refinement statistics			
*R*/*R*_free_^b^	0.2228/0.2519		0.2299/0.2770
			
Models	FtsQ 58–260		2× FtsQ 58–260
	FtsB 64–87		2× FtsB 64–87
	7 waters		0 waters
Bond length RMSD (Å)^c^	0.009		0.012
Bond angle RMSD (°)	1.162		1.637
Favored (%)^d^	99.5		99.5
Disallowed (%)^d^	0.0		0.5
MOLPROBITY score	97th percentile		98th percentile

PDB IDs	6H9N		6H9O

a Values in parentheses for these parameters refer to the highest recorded resolution shell.

b Five percent of the reflections were randomly selected before refinement.

c RMSD, root mean square deviation.

d Percentage of residues in the Ramachandran plot (PROCHECK “most favored” and “additionally allowed” added together).

### The FtsB 64–87 peptide is necessary and sufficient for the FtsQ-FtsB interaction.

**FIG 3:**
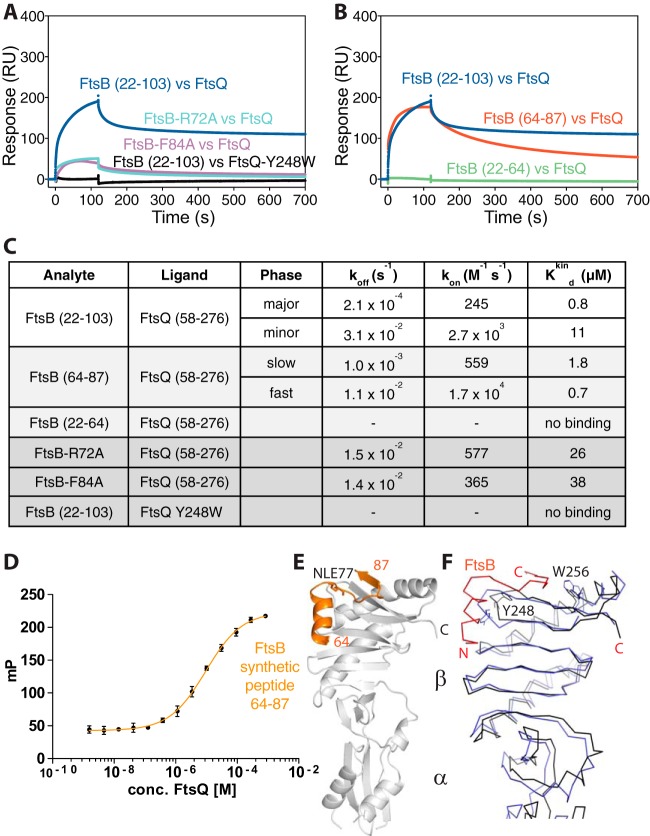
Validation of the crystal structure. (A) SPR experiments showing that FtsB mutants R72A and F84A display significantly compromised binding to FtsQ, as does FtsQ mutant Y248W to FtsB, which showed no binding. The crystal structure of the complex of FtsB and FtsQ implicates FtsB F84 and FtsQ Y248 in forming the binding interface. FtsB R72 is involved in a key salt bridge with FtsB E82, and its interruption seems to abrogate binding between FtsB and FtsQ as well. Note that these and other mutants were also investigated *in vivo* as described in the legends to Fig. 4 and Fig. S3 to S5 in the supplemental material. (B) SPR experiments investigating the role of the FtsB residues that were not resolved in the crystal structure, as only residues 64 to 87 were visible (residues 22 to 63 were not visible). FtsB binding to FtsQ requires only amino acids from residue 64 onwards until residue 87, and it is to be concluded that the remainder of the protein in the crystals is disordered. (C) Summary table quantifying the SPR data in panels A and B. (D) Corroborating the point that only FtsB residues 64 to 87 are needed for the interaction between FtsB and FtsQ, a fully synthetic peptide was produced (see Fig. S2), and its binding to FtsQ was investigated by fluorescence polarization, as the peptide also carried an FITC moiety at the N terminus. Fluorescence polarization (mP) is shown on the *y* axis. Fitting of the binding curve yielded a *K*_*d*_ of 9.5 µM, similar to the values obtained with recombinant FtsB 22–103 and 64–88 in panels B and C. (E) In fact, using a fully synthetic peptide of FtsB 22–87 (without FITC) produced crystals that, although in a different space group, show exactly the same structure and arrangement as observed before when adding the entire periplasmic domain of FtsB (22–103) to the FtsQ periplasmic domain. (F) Superposition of unbound E. coli FtsQ periplasmic domain (PDB ID 2VH1) (19) and our FtsQ structure in complex with FtsB. Overall, there are only small deviations, but Y248 dramatically changes its side chain conformer (and the entire loop 247–252 changes conformation slightly), as does W256.

First, we aimed at an *in vitro* validation of the crystal structure by investigating structure-guided mutant FtsB proteins and their binding to FtsQ (Fig. 3A and C). We chose FtsB R72A that forms a salt bridge within FtsB with E82 and is in direct contact with F84A that is positioned next to Y248 on the surface of FtsQ (Fig. 2D). Both mutant FtsB proteins showed approximately 10-fold reduced binding to immobilized FtsQ. Even more convincingly, FtsQ Y248W, a somewhat conservative mutation, showed no binding whatsoever when FtsB was tested. FtsQ Y248 (with A253) forms the central hydrophobic patch that FtsB latches onto (Fig. 2D). All mutants tested confirmed the binding mode of FtsB to FtsQ as shown by the crystal structure of the complex (Fig. 2), and this is further supported by an analysis of sequence conservation across FtsB homologues as depicted in Fig. S1 in the supplemental material; Fig. S1 shows strong conservation for residues shown in the crystal structure to interact with FtsQ.

10.1128/mBio.01346-18.1 FIG S1  Sequence conservation-based coloring of FtsB surface, shown in stereo. Around 100 sequences similar to E. coli FtsB (database ID FTSB_ECOLI) were identified using BLAST, and sequence conservation was determined from a CLUSTAL OMEGA multiple-sequence alignment with CONSURF (http://consurf.tau.ac.il/2016/). FtsB residues 64 to 87 are shown as a stick model colored by conservation from blue (most conserved residues in the alignment) to red (least conservation). FtsQ is shown as a gray molecular surface. All residues involved in major interactions with FtsQ are conserved in FtsB, in addition to internal interactions, such as R72-E82. Download FIG S1, PDF file, 0.2 MB. Copyright © 2018 Kureisaite-Ciziene et al. 2018 Kureisaite-Ciziene et al. This content is distributed under the terms of the Creative Commons Attribution 4.0 International license.

Resolving only residues 64 to 87 in the crystal structure of the FtsQ-FtsB complex raised the question of what the remaining residues present during crystallization do. For this, we went back to SPR and tested two FtsB subdomains comprising residues 22 to 64 and 64 to 87, chemically synthesized as peptides (Fig. S2, replacing methionine 77 with norleucine). SPR analysis unequivocally showed that the region within FtsB that is N terminal to the structure, residues 22 to 64, does not bind to FtsB, whereas the region containing only the ordered parts of the structure, residues 64 to 88, produced binding curves and binding parameters very similar to the original periplasmic 22–103 construct (construct consisting of residues 22 to 103) (Fig. 3B and C). The binding of the 64–87 peptide (peptide consisting of residues 64 to 87) was further investigated by fluorescence polarization, a solution assay. For this, fluorescein isothiocyanate (FITC)-labeled FtsB 64–87 peptide was synthesized (Fig. S2), and the decrease in polarization was measured while FtsQ was added (Fig. 3D). Because the resulting *K*_*d*_ of ~9 µM is lower (binding less tight) than what we measured by SPR and since identical reagents were used, these results give an impression of the variations caused by using different assay technologies but confirm in principle that FtsB 64–87 binds FtsQ in solution. To further show this, we crystallized an acetylated version of this peptide with FtsQ, resulting in essentially the same structure as observed for FtsB 22–103, despite being in a different crystallographic space group (Fig. 3E and Table 1). It is noteworthy that the synthesized FtsB 64–87 peptide is highly soluble in water, at least to 10 mM.

10.1128/mBio.01346-18.2 FIG S2  The table at the top shows peptide characteristics {molecular formula, one-letter code sequence, molecular weight, calculated and detected *m*/*z* ratios for multiple charged mass spectrometry ions ([M+nH]^n+^)}. Abbreviations: Ac, acetyl; FITC, fluorescein isothiocyanate fluorophore; PEG, 8-amino-3,6-dioxaoctanoyl linker; Nle, norleucine amino acid replacing methionine 77. (A) HPLC chromatogram at 210 nm of the peptide used for cocrystallization with FtsQ (Fig. 3D). The gradient was 20 to 50% ACN in 10 min, The mass spectrum of the corresponding peptide is shown. (B**)** HPLC chromatogram at 210 nm of FITC PEG peptide for fluorescence polarization (Fig. 3D). The gradient was 30 to 60% ACN in 10 min, starting at 3 min. The mass spectrum of the corresponding peptide is shown**.** Download FIG S2, PDF file, 0.1 MB. Copyright © 2018 Kureisaite-Ciziene et al. 2018 Kureisaite-Ciziene et al. This content is distributed under the terms of the Creative Commons Attribution 4.0 International license.

As already mentioned, FtsB binding to FtsQ involves only a few and minor changes to the conformation of FtsQ compared to a previous unbound structure of FtsQ (PDB ID 2VH1) (Fig. 3F) (19). Two exceptions are that FtsQ Y248 and W256 change their side chain conformations significantly upon binding, which fits well with our data showing that FtsQ Y248 is absolutely critical for the interaction with FtsB. The tyrosine side chain of Y248 is protruding from the main structure, whereas upon interaction with FtsB, it shifts deeply inward, providing aromatic stacking with FtsB F84 and potential hydrogen bonding to FtsB R72.

We conclude that binding of the periplasmic domain of FtsB to FtsQ in the absence of FtsL involves only FtsB residues 64 to 87 as shown by the structure, and this interaction can be faithfully reconstituted by using synthesized and water-soluble peptides comprising FtsB residues 64 to 87.

### The FtsQ-FtsB interaction in the context of bacterial cell division.

**FIG 4:**
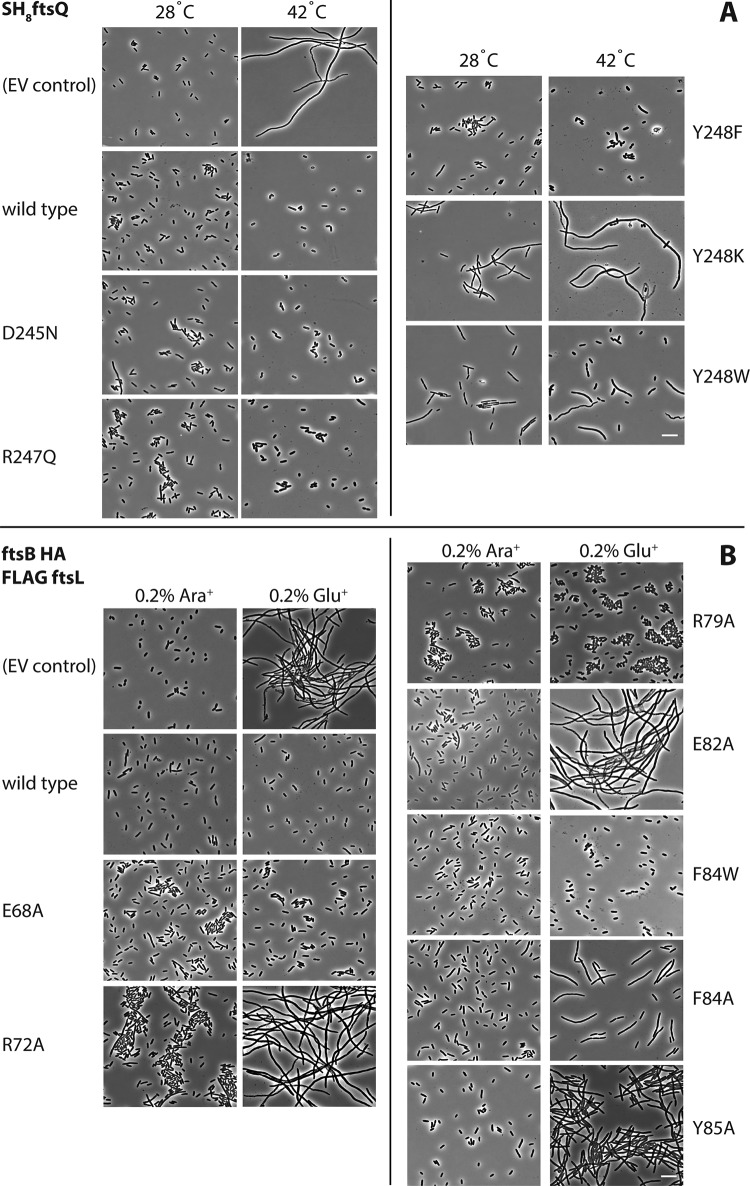
Mutating residues implicated in FtsB and FtsQ complex formation impairs cell division. (A) FtsQ mutants. The FtsQ temperature-sensitive E. coli strain LMC531 (40), harboring a plasmid for the expression of mutant SH_8_FtsQ was grown for 5 h at the permissive temperature (28°C) or at the nonpermissive temperature (42°C). Cells were analyzed by phase-contrast microscopy. EV, empty vector/plasmid. (B) FtsB mutants. The E. coli FtsB depletion strain NB946 (41), harboring a plasmid for the expression of mutant FtsB-HA (hemagglutinin-tagged FtsB) derivatives was grown under nondepleting conditions in the presence of 0.2% l-arabinose (Ara^+^) and under depleting conditions in the presence of 0.2% l-glucose (Glu^+^). Cells were analyzed by phase-contrast microscopy. EV, empty vector/plasmid. The mutated residues are highlighted in Fig. 2D. Bars, 10 µm.

On the basis of the FtsQ-FtsB structure (Fig. 2), we investigated E. coli cells harboring FtsQ and FtsB mutant proteins in order to validate the structure and to understand the contributions of various parts of the interface to the ability of the cell to divide. Functioning of the mutants was tested by low-level uninduced expression in E. coli strain LMC531, an *ftsQ* temperature-sensitive mutant. Cells grown at the permissive temperature were imaged by phase-contrast microscopy (Fig. 4A) or spotted onto solid medium (Fig. S3A), followed by incubation at the permissive and nonpermissive temperatures. Under these conditions, phase-contrast microscopy of LMC531 cells harboring the empty vector (EV) showed filamentation upon growth in liquid LB medium at the nonpermissive temperature, whereas positive-control cells harboring a tagged but otherwise wild-type FtsQ construct showed normal cells also at the nonpermissive temperature (Fig. 4A). Also, in the spot assay, the negative-control cells harboring the empty vector grew to high dilutions only at the permissive temperature, whereas the positive-control cells showed good growth at high dilution at the nonpermissive temperature (Fig. S3A).

10.1128/mBio.01346-18.3 FIG S3  (A) Spot assay of FtsQ mutants used in Fig. 4A. (B) Western blot showing expression levels of FtsQ mutants used in panel A. Download FIG S3, PDF file, 0.1 MB. Copyright © 2018 Kureisaite-Ciziene et al. 2018 Kureisaite-Ciziene et al. This content is distributed under the terms of the Creative Commons Attribution 4.0 International license.

Using both assays, cells with mutant FtsQ Y248F showed full complementation, indicating that the hydroxyl moiety in the tyrosine side chain is not required for functioning. In contrast, cells with FtsQ Y248K and even a Y248W substitution were unable to grow on plates at the nonpermissive temperature and showed a strongly filamentous phenotype using microscopy at the nonpermissive temperature and even at the permissive temperature, indicating a dominant-negative effect on cell division. We also investigated FtsQ D245N and R247Q, conservative mutations probing the role of the polar interactions of FtsQ with FtsB’s N-terminal portion of the binding region, and these mutations showed no effects. FtsQ mutants S250A, G251A, and W256A also showed no obvious effects in the spot assay, ruling out their significance for the FtsB interaction (Fig. S3A). In order to demonstrate that the phenotypes observed were not due to reduced protein levels of the mutated proteins, we performed Western blotting (Fig. S3B). Given the central role of Y248 and its very strong and selective phenotypes depending on the replacing residue type, we also investigated the localization of a fluorescently labeled version of FtsQ Y248W. This mutant protein showed strong cell division inhibition in both assays but localized normally in cells, being recruited correctly to division sites (Fig. S4), indicating that only downstream divisome interactions were affected, as predicted.

10.1128/mBio.01346-18.4 FIG S4  Fluorescence microscopy localization of FtsQ (Y248W) mutant protein in E. coli cells. Phase-contrast images, fluorescent images, and fluorescence profiles per cell (from 0 to 200 arbitrary units [AU]) plotted against normalized cell length (from 0 to 100%) of temperature-sensitive FtsQ LMC531 strain expressing wild-type FtsQ or FtsQY248W fused to mNeonGreen (mNG) at 30 or 42°C. *n* is the number of cells. The graphs show the average total fluorescence per cell plotted against the normalized cell length. Therefore, filaments have a higher fluorescence per cell. In addition, the levels of expression of the proteins are higher at the nonpermissive temperature. Bar, 2 µm. Download FIG S4, PDF file, 1.1 MB. Copyright © 2018 Kureisaite-Ciziene et al. 2018 Kureisaite-Ciziene et al. This content is distributed under the terms of the Creative Commons Attribution 4.0 International license.

The central role of FtsQ Y248 in the interaction interface suggests that specific mutations at this position are not tolerated because they affect the interaction with FtsB. If this is the case, complementary mutations in this area of the FtsQB complex in FtsB should have a similar effect. To examine this, structure-guided FtsB mutant proteins were tested for functionality by low-level background expression in a conditional E. coli FtsB mutant strain NB946 in which the chromosomal *ftsB* gene is under control of an arabinose promoter. Depletion of FtsB occurs when 0.2% l-arabinose is replaced by 0.2% l-glucose in the growth medium leading to filamentation, and eventually cell death, due to the essential nature of FtsB as observed by phase-contrast microscopy and a spot assay (empty vector [EV] negative control [Fig. 4B; Fig. S5]). It also confirmed that a hemagglutinin (HA)-tagged FtsB construct that was used as the basis for the mutagenesis was able to complement growth and proper cell division in cells grown in the absence of the inducer l-arabinose in contrast to NB946 cells harboring the empty vector (Fig. 4B; Fig. S5). Mutations were introduced changing conserved residues within the FtsB domain binding to FtsQ and analyzed using the microscopy and spot assay described above for FtsQ mutants. FtsB F84A, also compromised in the SPR assay (Fig. 3A and C), appeared nonfunctional, although cell filamentation was relatively mild, suggesting that the aromatic interaction with FtsQ Y248 is critical for FtsB functioning. In agreement with this supposition, changing F84 into tryptophan did not affect FtsB functioning. Y85A was also nonfunctional with a rather strong filamentation phenotype that was even more pronounced and dominant in a double mutant (F84A Y85A) (spot assay only [Fig. S5]).

10.1128/mBio.01346-18.5 FIG S5  Spot assay showing FtsB mutants used in Fig. 4B. Download FIG S5, PDF file, 0.1 MB. Copyright © 2018 Kureisaite-Ciziene et al. 2018 Kureisaite-Ciziene et al. This content is distributed under the terms of the Creative Commons Attribution 4.0 International license.

The loop between the α-helix and β-strand of the FtsB domain that is resolved in the FtsQB complex structure is connected via two salt bridges, R72-E82 and E68-R79 (Fig. 2D). In agreement with the SPR data (Fig. 3A and C), both R72A and E82A did not complement FtsB depletion and cells with these two mutations showed a strong filamentation phenotype, suggesting that this salt bridge is essential for interaction or possibly for shaping FtsB into the correct fold for binding. In contrast, FtsB E68A and R79A appeared fully functional, suggesting that this second salt bridge is largely dispensable. These findings are further supported by Fig. S1, which shows that the R72-E82 salt bridge is more conserved than E68-R79. Similarly, FtsB E65 and E69, potentially interacting with FtsQ R196, could be changed into alanine without functional consequences (spot assay only [Fig. S5]). Because of the very low level of FtsB in cells and the low protein levels needed to complement in our assays, we have been unable to test protein levels in cells (Fig. 4B and Fig. S5) reliably by Western blotting.

## DISCUSSION

It has been proposed that FtsL and FtsB form a coiled-coil/leucine zipper heterodimer that binds to FtsQ (22, 23). We show here that at least the periplasmic domains of E. coli FtsL and E. coli FtsB do not interact on their own, but both proteins bind well to FtsQ, forming a heterotrimeric complex. This has been reported before based on pulldown experiments (25). On the basis of these data and the lack of coiled-coil/leucine zipper prediction for the periplasmic domain of FtsL, we suggest that FtsL binds to both FtsB and FtsQ in the complex, at least in E. coli. It is worth noting that FtsL proteins from other organisms do show weak coiled-coil/leucine zipper predictions and FtsL from Bacillus subtilis shows strong prediction. It is conceivable that different interaction modes exist between these small proteins in different organisms. It is also conceivable that the transmembrane segments of FtsQ, FtsL, and FtsB make significant contributions to their interactions and different results would have been obtained in their presence.

The structure of the complex between the periplasmic domains of E. coli FtsB and FtsQ contained FtsB residues 64 to 87 only. We showed that this subdomain is indeed necessary for the interaction but is also sufficient, suggesting that the remaining residues within FtsB are disordered in the crystals and do not bind to FtsQ.

Overall, the interaction interface between FtsQ and FtsB observed here in the structure (Fig. 2) corresponds to the main interaction site that was identified by *in vivo* site-specific photo-cross-linking (24). In that study, 50 surface-exposed positions in the periplasmic domain of E. coli FtsQ were changed into *p*-benzoyl-l-phenylalanine (Bpa) using amber suppressor technology for photon-induced cross-linking of FtsQ with its binding partners. Using this scanning approach, the strongest cross-linking to FtsB was observed at Y248Bpa and S250Bpa. Moreover, paired cysteine mutagenesis enabled the formation of a disulfide bond between FtsQ S250C and FtsB V88C. Both findings are in line with our structure. Although FtsB 88V is not included in the structure, the last resolved residue, L87, is immediately juxtaposed to FtsQ S250, and Y248 in FtsQ is the central residue of the hydrophobic patch on FtsQ that binds FtsB. Similarly, functional analysis of the FtsQ Bpa mutants showed that they all complemented the function of FtsQ in an FtsQ Ts mutant grown at the nonpermissive temperature, except for Y248Bpa, highlighting again the crucial nature of this position that does not allow even small alterations. It is also notable that the orientation of the Y248 side chain is different in the unbound FtsQ (PDB ID 2VH1) (19) compared with the FtsQ-FtsB complex described here (Fig. 3F).

**FIG 5:**
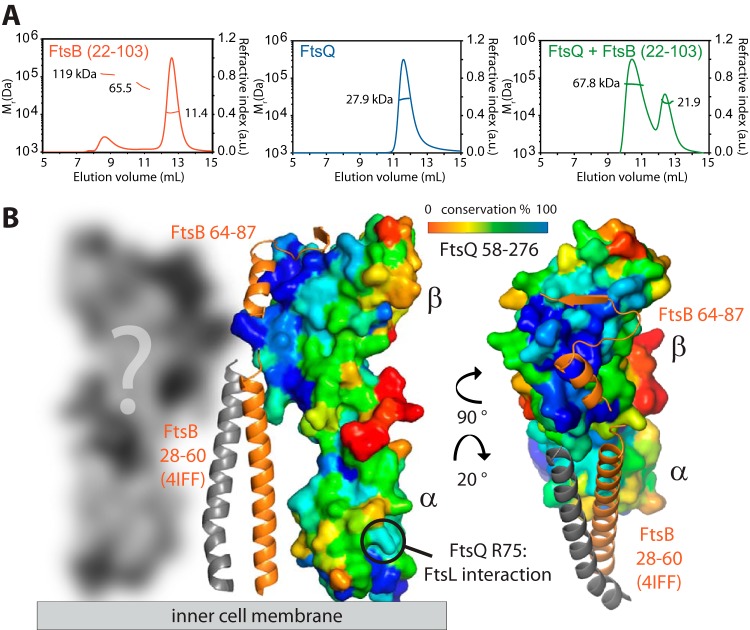
Model of the periplasmic complex between E. coli FtsB and FtsQ. (A) Size exclusion chromatography with multiple angle light scattering (SEC-MALS) of the complex of FtsB and FtsQ. FtsB forms large oligomers on its own. FtsQ is monomeric alone, but together, FtsB and FtsQ most likely form a 2 + 2 complex. Analytic ultracentrifugation (AUC) was also used to investigate the same complex with very similar results (Fig. S6). Relative molecular weight (*M*_r_) (in daltons) is shown on the left-hand *y* axis. Refractive index is shown on the right-hand *y* axis in arbitrary units (a.u.). (B) Residues 22 to 64 of FtsB were shown to not interact with FtsQ (Fig. 3B and C). A previous crystal structure (PDB ID 4IFF) (30) showed that residues 28 to 60 are able to form a coiled-coil arrangement with each other (although through artificial dimerization), and it is possible that this interaction leads to the dimerization of the FtsB and FtsQ complex into the observed 2 + 2 stoichiometry. Residues 22 to 64 within FtsB link its single transmembrane helix to the FtsQ-interacting domain in FtsB and also produce the putative dimer as shown by forming a homodimeric coiled coil. It was previously shown that a region around R75 in FtsQ links to FtsL (24). FtsQ is shown in surface representation with sequence conservation color coded (most conserved shown in blue and least conserved shown in red). It is clear from the plot that the FtsB binding region (residues 64 to 87) covers most of the highly conserved patch on the β domain of FtsQ.

Of note, FtsQ S250 and Y248 are part of a conserved region in the interaction interface with FtsB that stretches from Y243 to W256 (compare Fig. 2C and D with Fig. 5B). Single substitutions in this motif, D245N, R247Q, G251A, W256A (this study), G255C, and S250C (24) did not affect functioning of FtsQ consistent with the observed tolerance of FtsQ toward Bpa substitutions. In an independent study, FtsQ amino acids 257 to 276 were shown to be dispensable for function, whereas shorter truncations were less stable, possibly because FtsB and/or FtsL were not recruited (28).

In the FtsQ-FtsB structure, FtsB Y85 is oriented toward FtsQ L226, a position that was previously shown to cross-link very strongly to FtsB (24). In accordance with these data, it has been shown that FtsB truncated at Y85 is unable to complement and interact with FtsQ, whereas FtsB truncated at D90 is fully functional (29), and this is also supported by our data showing that FtsB 64–88 binds as well as the entire periplasmic domain comprising residues 22 to 103 (Fig. 3B and C).

The three residues upstream of FtsB R79 have been individually changed to cysteine (S76C, M77C, and T78C) in a previous study (24) without any effect on FtsB functioning, arguing that the precise sequence of the loop that connects the FtsB α-helix and β-strand is not very relevant for FtsB functioning.

Finally, we would like to speculate on the nature of the FtsQ-FtsB complex when in the membrane and with FtsL and FtsN. Here, we found that FtsQ and FtsB together form molecular species bigger than a 1:1 complex (Fig. 2A and B). In order to understand this better, we also performed size exclusion chromatography with multiple angle light scattering (SEC-MALS) and analytic ultracentrifugation (AUC) (Fig. 5A; see also Fig. S6A in the supplemental material). Size estimates from those experiments for FtsB, FtsQ, and FtsQ-FtsB are summarized in Fig. S6B. FtsB alone occurred in several species, including some high-molecular-weight oligomers. FtsQ was almost exclusively monomeric, and the FtsQ-FtsB complex showed two species, most likely corresponding to 1:1 and 2:2 complexes. Taking this into account and the previous structure of the coiled-coil segment of FtsB comprising residues 28 to 60 (PDB ID 4IFF) (30), a hybrid model of the entire periplasmic FtsQ-FtsB complex can be assembled (Fig. 5B). In this model, the FtsB coiled-coil segment, disordered in our crystal structure, forms a homodimer, as observed in the 4IFF crystal structure (that, admittedly, was artificially dimerized by fusing it to a coiled-coil dimerization domain) (30). FtsQ binds only to the C-terminal part of FtsB, containing residues 64 to 87 as demonstrated here by the structure and other experiments. Because FtsB is a dimer, this means that the complex will recruit two FtsQ molecules, explaining the observed 2:2 stoichiometry and a measured molecular mass of 68 to 69 kDa. The model also predicts that the transmembrane segments of FtsB are dimerized or in very close proximity, in contrast to those of the two FtsQ molecules, which could be further apart. As shown, FtsL binds together with FtsB to FtsQ, most likely binding to surfaces on FtsB and FtsQ simultaneously. It is not clear from looking at sequence conservation on the FtsQ surface where those binding surfaces are located since the FtsB binding covers almost perfectly a highly conserved patch on FtsQ (Fig. 5B, blue patch), although we would like to speculate that FtsL might bind to the second interaction hot spot on FtsQ around residue FtsQ R75 (24). Also, FtsN binding, demonstrated here biochemically, involves binding to FtsQ independently of FtsB, and the location of that binding site is currently unknown.

10.1128/mBio.01346-18.6 FIG S6  (A) Analytic ultracentrifugation (AUC) of FtsQ and FtsB alone and in complex. (B) Summary table of SEC-MALS (Fig. 5A) and AUC data (panel A) describing the properties of FtsQ, FtsB, and their complexes. Download FIG S6, PDF file, 0.1 MB. Copyright © 2018 Kureisaite-Ciziene et al. 2018 Kureisaite-Ciziene et al. This content is distributed under the terms of the Creative Commons Attribution 4.0 International license.

We conclude that our crystal structure is a valid representation of the complex formed during cell division and that it is consistent with previous data regarding FtsQ and FtsB. The complex represents an exciting target for structure-based design of protein interaction inhibitors shutting down cell division based on peptides or peptide mimetics, as the FtsQ-FtsB interaction surface is small, the 64–87 FtsB peptide is water soluble, and the interaction is easily interrupted by single mutations. In the future, it may be possible to biochemically add more components of the divisome as we report here direct interactions of FtsQ also with FtsL and FtsN and to investigate the structure and function of divisome subcomplexes in the current absence of any reports of isolated or overexpressed holo complexes, with only one study showing high-molecular-weight divisome complexes by Western blotting (31).

## MATERIALS AND METHODS

### Surface plasmon resonance.

Surface plasmon resonance (SPR) was performed using a Biacore T200 instrument using CM5 sensor chips (GE Healthcare). Both reference control and analyte channels were equilibrated in HBS-N buffer (0.01 M HEPES [pH 7.4], 0.15 M NaCl) at 20°C. FtsQ with residues 58 to 276 [FtsQ (58–276)] or FtsL with residues 58 to 121 [FtsL (58–121)] were immobilized onto chip surfaces through amide coupling using the supplied kit to reach resonance unit (RU) values of ~1,000 and 3,300, respectively.

Triplicate SPR runs were performed in 10 mM HEPES (pH 7.4), 150 mM NaCl, and 0.4% (wt/vol) glycerol with analytes injected for 120 s, followed by a 600-s dissociation in 1:2 dilution series with initial concentrations of FtsB (22–103) and FtsB (64–88) of 80 µM, FtsB (22–64) of 200 µM, FtsB-F84A and FtsB-R72A of 68 µM, FtsN (58–320) and FtsN (58–320) plus FtsB (22–103) of 40 µM each for experiments where FtsQ was the ligand. For runs where FtsL (58–121) was attached to the chip surface, initial concentrations of FtsQ (58–276) and FtsB (22–103) were 40 µM each either alone or in combination. The surface was regenerated with 10 mM HCl for 60 s. After reference and buffer signal correction, sensogram data were fitted using KaleidaGraph (Synergy Software) and Prism (GraphPad Software).

### Cloning, protein expression, and purification.

FtsQ (58–276) and FtsN (57–319) from Escherichia coli strain K-12 (FTSQ_ECOLI for FtsQ and FTSN_ECOLI for FtsN) were amplified using PCR from genomic DNA, cloned into the NdeI/BamHI sites of plasmid pHis17, and expressed as a C-terminal His_6_ tag in E. coli (see Table 1 for the exact protein sequences).

FtsB (22–103) and FtsL (58–121) from E. coli (FTSB_ECOLI for FtsB and FTSL_ECOLI for FtsL) were amplified using PCR from genomic DNA, cloned into the NdeI/BamHI sites of plasmid pET15b and expressed as a N-terminal His_6_ tag in E. coli (see Table 1 for the exact protein sequences).

Recombinant proteins were expressed in E. coli C41(DE3) cells, which were grown in 2× TY medium, consisting of 16 g Tryptone, 20 g yeast extract, 5 g NaCl, pH 7.4, with ampicillin (100 mg/liter) at 37°C with 200 rpm shaking to an optical density at 600 nm (OD_600_) of 0.6. The cultures were then shifted to 20°C and induced by the addition of 0.5 mM isopropyl-β-d-1-thiogalactopyranoside (IPTG) before overnight incubation. Selenomethionine-substituted FtsB (22–103) cell cultures were grown to early log phase (*A*_600_ of 0.6) at 37°C in M9 minimal medium, consisting of 5.8 g Na_2_HPO_4_, 3 g KH_2_PO_4_, 0.5 g NaCl, 1 g NH_4_Cl, and supplemented with 0.4% glucose and 2 mM MgSO_4_. One hundred milligrams/liter of dl-selenomethionine (Generon), 100 mg/liter of lysine, threonine, and phenylalanine, and 50 mg/liter of leucine, isoleucine, and valine were added as solids. Fifteen minutes later, protein expression was induced with 0.5 mM IPTG and grown overnight at 20°C.

### Protein purification.

For crystallization experiments, bacterial cell pellets containing overexpressed FtsQ (58–276) and FtsB (22–103) were mixed and resuspended in 50 mM Tris, 150 mM NaCl, 10% glycerol, pH 8.0. Cell lysis was carried out at 25,000 lbs/in^2 ^using a cell disruptor (Constant Systems), and the lysate was clarified by centrifugation at 20,000 rpm for 30 min at 4°C. The supernatant was passed over a HisTrap high-performance (HP) column (GE Healthcare). The column was equilibrated with 25 mM Tris, 150 mM NaCl, 10% glycerol, 0.5 mM dithiothreitol (DTT), pH 8.0. Proteins were eluted with 300 mM imidazole in the same buffer. Peak fractions were concentrated and loaded onto a HiLoad Sephacryl S300 16/60 column (GE Healthcare) equilibrated in 25 mM Tris, 100 mM NaCl, 2 mM DTT, pH 7.4. Purified FtsQB complex was concentrated to 6 mg/ml using centrifugal concentrators (Vivaspin; Sartorius) for immediate use. For SPR and size exclusion chromatography with multiple angle light scattering (SEC-MALS) assays, FtsB, FtsL, FtsQ, and FtsN proteins were purified separately using the same protocol and then concentrated to 5 to 10 mg/ml before freezing in liquid nitrogen and storage at −80°C.

### Crystallization, data collection, and structure determination.

Crystallization conditions were found using our in-house high-throughput crystallization platform (32) by mixing 200 nl selenomethionine (SeMet)-substituted FtsBQ or wild-type FtsQB solution at 6 mg/ml with 200 nl of 1,920 different crystallization reagents in MRC vapor diffusion sitting drop plates. Crystals were grown at 19°C by vapor diffusion in 0.15 M potassium thiocyanate, 20% PEG 550 MME (polyethylene glycol 500 methyl ether), 0.1 M sodium cacodylate (pH 6.5).

For the FtsQ (58–276)/FtsB (64–88 peptide) complex crystallization, FtsB synthetic peptide was added to 4 mg/ml FtsQ protein solution at a molar ratio of 1 FtsQ to 4 FtsB. Crystals were grown at 19°C by vapor diffusion in 0.057 M potassium thiocyanate, 10% PEG 550 MME, 0.1 M sodium cacodylate (pH 6.5). Glycerol (25%, vol/vol) was used as a cryoprotectant. Conditions yielding crystals were optimized, and crystals from either the initial screens or subsequent optimizations were selected for data collection. Diffraction images were collected from single frozen crystals at beamlines I03, I04 and I04-1 at Diamond Light Source (Harwell, UK) as indicated in Table 1. Diffraction images were processed with XDS (33) and further processed with the CCP4 package of programs (34). Initial phases were determined by molecular replacement using PHASER with a previous E. coli FtsQ structure as the search model (PDB ID 2VH1) (35). Iterative model building and refinements were carried with MAIN, REFMAC, and PHENIX (36–38). Data and model statistics are summarized in Table 1. 

### FtsB (64–87) peptide synthesis, purification, and characterization.

Peptides were synthesized by 9-fluorenylmethoxy carbonyl (Fmoc)-based solid-phase peptide synthesis on H-rink amide ChemMatrix resin (Merck). The peptide sequence was assembled using an automated synthesizer (Syro II; MultiSynTech). For amino acid coupling, 4 equivalents (eq) of the Fmoc-protected amino acids (Iris Biotech) according to the initial loading of the resin were mixed with 4 eq of 1-[bis(dimethylamino)methylene]-1*H*-1,2,3-triazolo[4,5-*b*]pyridinium 3-oxid hexafluorophosphate (HATU) and 8 eq of *N*,*N*-diisopropylethylamine (DIPEA) and added to the resin for 40 min. In a second coupling step, the resin was treated with 4 eq of the Fmoc-protected amino acid mixed with 4 eq of benzotriazole-1-yl-oxy-tris-pyrrolidino-phosphonium hexafluorophosphate (PyBOP) and 8 eq of 4-methylmorpholine (NMM) for 40 min. After double coupling, a capping step to block free amines was performed using acetanhydride and DIPEA in *N*-methyl-2-pyrrolidinone (NMP) (1:1:10) for 10 min. Fmoc deprotection was performed twice using 20% piperidine in dimethylformamide (DMF) for 5 min each time. After each step, the resin was washed four times with DMF. For the crystallization experiment of FtsB 64–87 with FtsQ, the peptide was acetylated with acetic anhydride (Ac_2_O) final Fmoc deprotection. The fluorescently labeled peptide for affinity measurements was N-terminally coupled with 8-(9-fluorenylmethyloxycarbonyl-amino)-3,6-dioxaoetanoic acid as stated above, and subsequently fluorescein isothiocyanate (FITC) was coupled manually twice using 8 eq of DIPEA for 1.5 h each time. Final cleavage was performed twice with 94% trifluoroacetic acid (TFA), 2.5% 1,2-ethanedithiole (EDT), 2.5% H_2_O, and 1% triisopropylsilane (TIPS) for 1.5 h each time. The cleavage solutions were combined, and peptides were precipitated with diethyl ether (Et_2_O) at −20°C for 10 min. The peptides were resolved in water-acetonitrile (ACN) at a 5:5 ratio and purified by reversed-phase high-performance liquid chromatography (HPLC) (Nucleodur C_18_ column [Macherey-Nagel]; 10 by 125 mm; 110 Å; 5-µm particle size) using a flow rate of 6 ml/min (solution A is ACN with 0.1% TFA and solution B is water with 0.1% TFA). Obtained pure fractions were pooled and lyophilized. Peptide characterization was performed by analytic HPLC (1260 Infinity [Agilent Technology]; flow rate of 1 ml/min, solutions A and B as described above) coupled with mass spectrometry (6120 Quadrupole LC/MS mass spectrometer [Agilent Technology]) using electrospray ionization (Eclipse XDB-C_18_ column [Agilent]; 4.6 by 150 mm; 5-µm particle size). Analytic HPLC chromatograms recorded at 210 nm are shown in Fig. S2 in the supplemental material. Quantification of acetylated peptide was performed by HPLC-based comparison (chromatogram at 210 nm) with a reference peptide, and quantification of fluorescein-labeled peptide was performed using the extinction coefficient ε = 77.000 M^−1^ cm^−1^ of FITC in 100 mM sodium dihydrogen phosphate, pH 8.5.

### Fluorescence polarization assay.

To determine the affinity of the FtsB peptide to FtsQ, a 0.1 mM dimethyl sulfoxide (DMSO) solution of the FITC-labeled peptide was dissolved in 10 mM HEPES (pH 7.4), 150 mM NaCl, and 0.1% Tween 20 to yield a 40 nM peptide solution. A threefold dilution of FtsQ (15 µl per well) was presented in a 384-well plate (black, flat bottom [Corning]) and incubated with the peptide solution (5 µl; final peptide concentration, 10 nM). The final protein concentration ranged from 800 µM to 0.5 nM. After incubation for 1 h at room temperature, fluorescence polarization was measured using a Tecan Spark 20M plate reader with an excitation wavelength (λ_ex_) of 485 nm and λ_ex _of 525 nm. *K*_*d*_ (dissociation constant) values were determined by nonlinear regression analysis of dose-response curves using GraphPad Prism software.

### Size exclusion chromatography with multiangle light scattering.

The masses in solution of FtsB (22–103) and FtsQ (58–276) both singly and in a complex were estimated using an online Dawn Heleos II 18 angle light scattering instrument (Wyatt Technologies) coupled to an Optilab rEX online refractive index detector (Wyatt Technologies). Protein samples (100 µl) were resolved on a Superdex S-75 10/300 analytic gel filtration column (GE Healthcare), preequilibrated with 25 mM Tris (pH 7.4), 100 mM NaCl, and 1 mM DTT at 0.5 ml/min. Protein concentration was determined from the excess differential refractive index based on dn/dc (slope of refractive index against concentration) of 0.186 mg/ml. The light scattering and protein concentration at each point across the peaks in the chromatograph were used to determine the absolute molecular mass from the intercept of the Debye plot using Zimm’s model as implemented in the ASTRA version 5.3.4.20 software (Wyatt Technologies). In order to determine the interdetector delay volumes, band broadening constants, and the detector intensity normalization constants for the instrument, we used bovine serum albumin (BSA) as a standard prior to sample measurements.

### Analytic ultracentrifugation.

Samples of FtsB (22–103) and FtsQ (58–276) alone or mixed together at concentrations of 1 mg/ml were subjected to velocity sedimentation at 50,000 rpm at 20°C in 25 mM Tris (pH 7.4), 100 mM NaCl, and 1 mM DTT using 12-mm double sector cells in an An50Ti rotor using an Optima XL-I analytic ultracentrifuge (Beckmann). The sedimentation coefficient distribution function, c(s), was analyzed using the SEDFIT program, version 15.0 (39). The partial-specific volumes (v-bar), solvent density, and viscosity were calculated using SEDNTERP software (Thomas Laue, University of New Hampshire).

### Plasmids, strains, and growth conditions for complementation assays.

E. coli strains BL21(DE3), LMC531 [*ftsQ1*(Ts)] (40), and NB946 (41) were grown in LB medium with shaking at 200 rpm. When indicated, strains LMC531 and NB946 were grown in GB1 medium [4.83 g of K_2_HPO_4_·3H_2_O, 2.95 g of KH_2_PO_4_, 1.05 g of (NH_4_)_2_SO_4_, 0.10 g of MgSO_4_·7H_2_O, 0.28 mg of FeSO_4_·7H_2_O, 7.1 mg of Ca(NO_3_)_2_·4H_2_O, 4 mg of thiamine, 0.4% glycerol (vol/vol), 0.2% Casamino Acids (wt/vol) per liter at pH 7.0]. When required, 0.2% l-arabinose, 0.2% l-glucose, 100 µg/ml ampicillin, 30 µg/ml chloramphenicol, 25 µg/ml kanamycin, and 50 µg/ml spectinomycin were added to the culture medium.

Standard PCR and cloning techniques were used for DNA manipulation. p29SENX-SH8FtsQ encoding full-length FtsQ with an amino-terminal StrepII-His8 dual tag (24) was used as the template for mutagenesis in *SH8ftsQ* using overlap extension PCR.

The DNA sequence encoding full-length FtsB with a carboxy-terminal hemagglutinin (HA) tag and the DNA sequence encoding full-length FtsL with an amino-terminal FLAG tag were cloned by PCR and transferred into the pCDF plasmid (Novagen) resulting in pCDF-FtsB-HA FLAG-FtsL. Mutations in *ftsB-HA* were introduced by overlap extension PCR.

Plasmids pTHV037-mNG-FtsQ was constructed by cloning *ftsQ* into pTHV037 (42). *ftsQ* was amplified from E. coli genomic DNA with primers containing restriction sites for EcoRI and NcoI, also used to digest pTHV037. A triple asparagine linker was introduced immediately downstream of the EcoRI site. The plasmid and insert were ligated with T4 DNA ligase (NEB, Ipswich, MA). Finally, the plasmid was amplified by circular PCR to incorporate the Y248W mutation and obtain the plasmid pTHV037-mNG-FtsQY248W.

### Functionality and detection of FtsQ and FtsB mutants.

To assess functioning of FtsQ mutants by microscopy, E. coli LMC531 cells harboring one of the p29SENX-SH8FtsQ mutants were grown overnight at 28°C in LB medium supplemented with 100 µg/ml ampicillin. The same medium was inoculated 1:100 with the overnight cultures and incubated for 5 h at 28°C or 42°C. To assess functioning of FtsB mutants by microscopy, E. coli NB946 cells harboring one of the pCDF-FtsB-HA FLAG-FtsL mutants were grown overnight at 37°C in LB medium supplemented with 25 µg/ml ampicillin, 20 µg/ml kanamycin, and 0.2% l-arabinose. Then, LB medium supplemented with the indicated antibiotics and 0.2% l-glucose or 0.2% l-arabinose was inoculated 1:100 with the overnight cultures and incubated for 5 h at 37°C. The cells were fixed by the addition of formaldehyde to 3% at room temperature for 15 min, harvested by centrifugation (13,000 × *g*, 2 min), and resuspended in phosphate-buffered saline (PBS) for the examination of cell morphology by phase-contrast microscopy. To determine the expression of mutant FtsQ under these conditions, cells were harvested from the cultures by centrifugation (13,000 × *g*, 2 min), and the pellet was resuspended in 2× SDS sample buffer and incubated for 5 min at 96°C. The crude cell lysates of *E*. *coli* LMC531 were separated on 12% SDS-polyacrylamide gels and analyzed by Western blotting using anti-FtsQ and anti-FtsB affinity-purified rabbit polyclonal antibodies.

To assess functioning of FtsQ mutants by complementation of growth on solid medium, E. coli LMC531 cells harboring one of the p29SENX-SH8FtsQ mutants were grown overnight at 28°C in LB medium with 100 µg/ml ampicillin. The same medium was inoculated 1:100 with the overnight cultures, incubated for 10 h at 28°C, and then diluted 1:100 in GB1 medium with 100 µg/ml ampicillin for overnight growth at 28°C. The same medium was inoculated 1:100 with the overnight cultures and incubated for 5 h at 28°C or 42°C. The cultures were 10-fold serially diluted, and 4 µl of each dilution was spotted on GB1 agar medium. Growth was assessed after 21-h incubation at 28°C or 18-h incubation at 42°C.

To assess functioning of FtsB mutants by complementation of growth on solid medium, E. coli NB946 cells harboring one of the pCDF-FtsB-HA mutants were grown overnight at 37°C in LB medium supplemented with 25 µg/ml ampicillin, 20 µg/ml kanamycin, and 0.2% l-arabinose. The same medium was inoculated 1:100 with the overnight cultures, incubated for 10 h at 37°C, and then diluted 1:100 in the same GB1 medium for overnight growth at 37°C. The same medium was inoculated 1:100 with the overnight cultures and incubated for 5 h at 37°C. The bacterial suspensions were diluted and spotted as described above on GB1 agar medium supplemented with the indicated antibiotics and either 0.2% l-arabinose or 0.2% l-glucose, and growth was analyzed after 18-h incubation at 37°C.

### Fluorescence microscopy.

The temperature-sensitive FtsQ strain LMC531 (40) containing plasmid pTHV037-mNG-FtsQ or pTHV037-mNG-FtsQY248 was grown in LB overnight at 30°C supplemented with 100 µg/ml ampicillin. The day after, samples were diluted (1:500) in 25 ml of the same medium and grown at 30°C until the OD_600_ was 0.25. The cells were then diluted 1:10 in cultures at 30 or 42°C. When the OD_600_ was 0.05, samples were induced with 15 µM IPTG for 2 mass doubling times. Noninduced cell samples were used as the control. Cells were immobilized on 1% agarose (43) and imaged with a Nikon Eclipse T1 microscope (Nikon Plan Fluor 100×/1.30 oil ph 3 DLL objective) coupled to an electron-multiplying charge-coupled-device (EMCCD) camera (Hamamatsu Flash 4.0).

### Accession number(s).

Atomic coordinates have been deposited in the Protein Data Bank (PDB) with identifiers 6H9N and 6H9O.
