# Supplementary figures and images for: Structural Analysis of the Interaction between the Bacterial Cell Division Proteins FtsQ and FtsB
Source: mBio. 2018 Sep 11;9(5):e01346-18. doi: 10.1128/mBio.01346-18 (PMC6134095; doi:10.1128/mBio.01346-18)

Supplementary Figure S1.

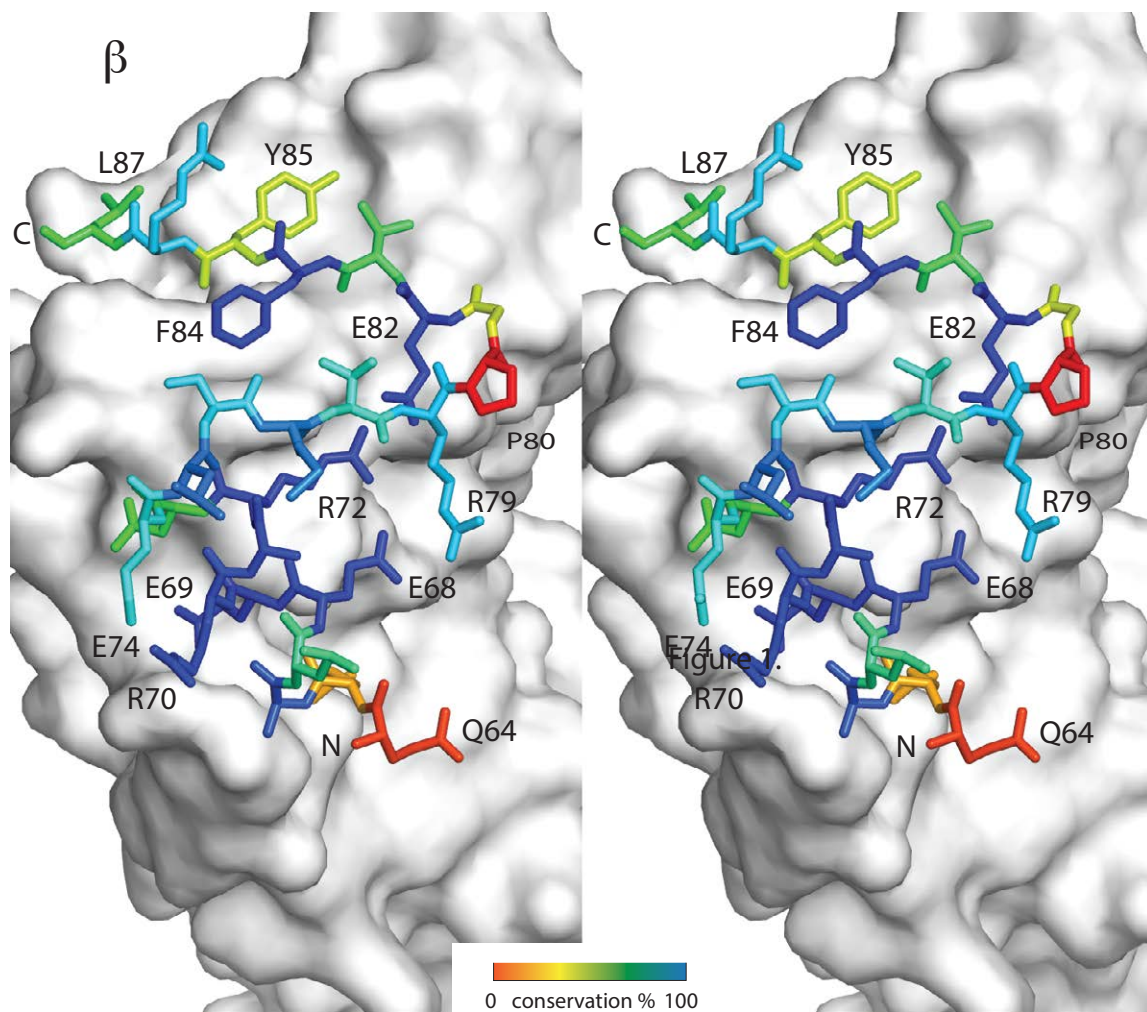

Supplement: FIG S1 [file mbo004184054sf1.pdf]

Supplementary Figure S3.

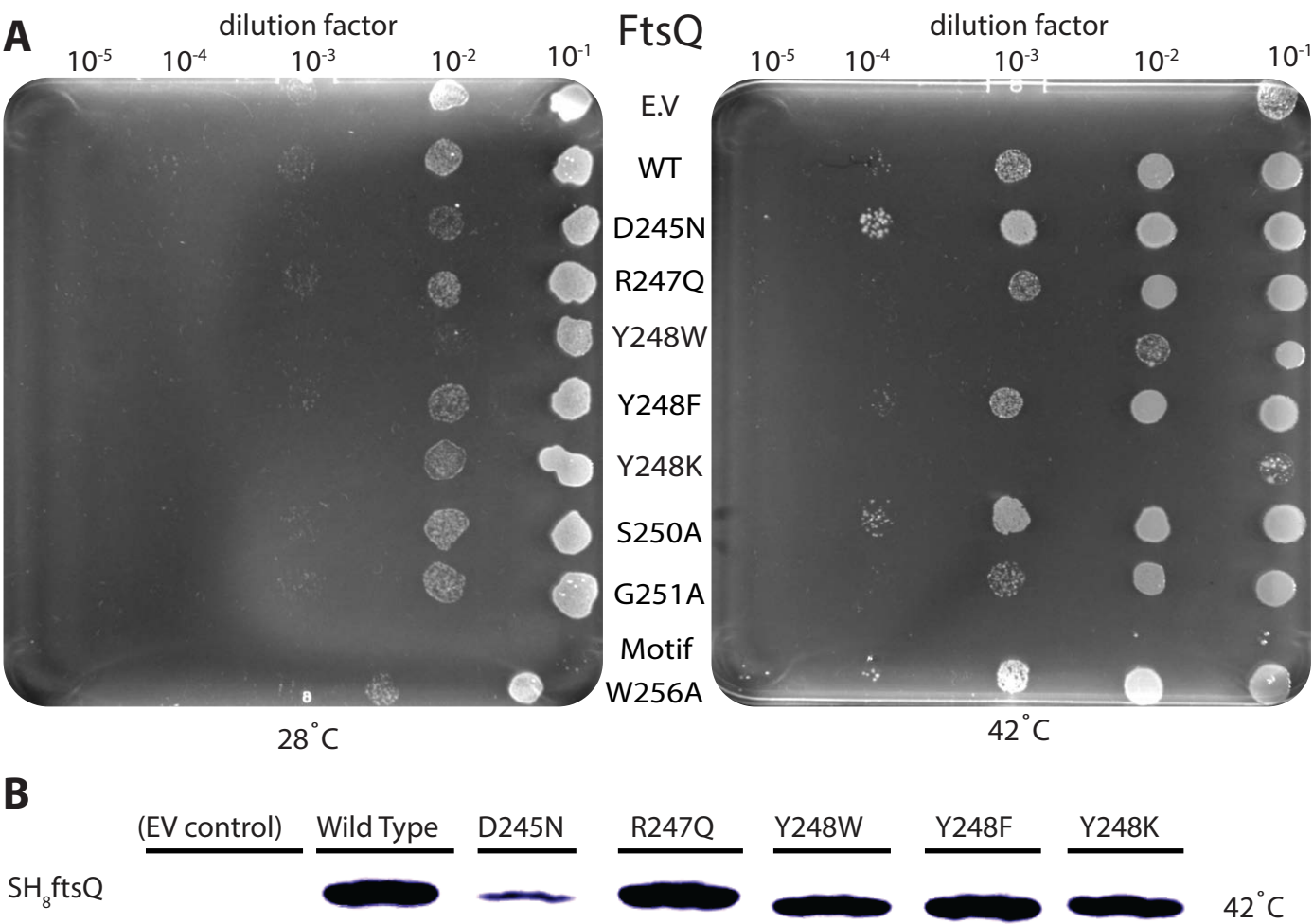

Supplement: FIG S3 [file mbo004184054sf3.pdf]

Supplementary Figure S4.

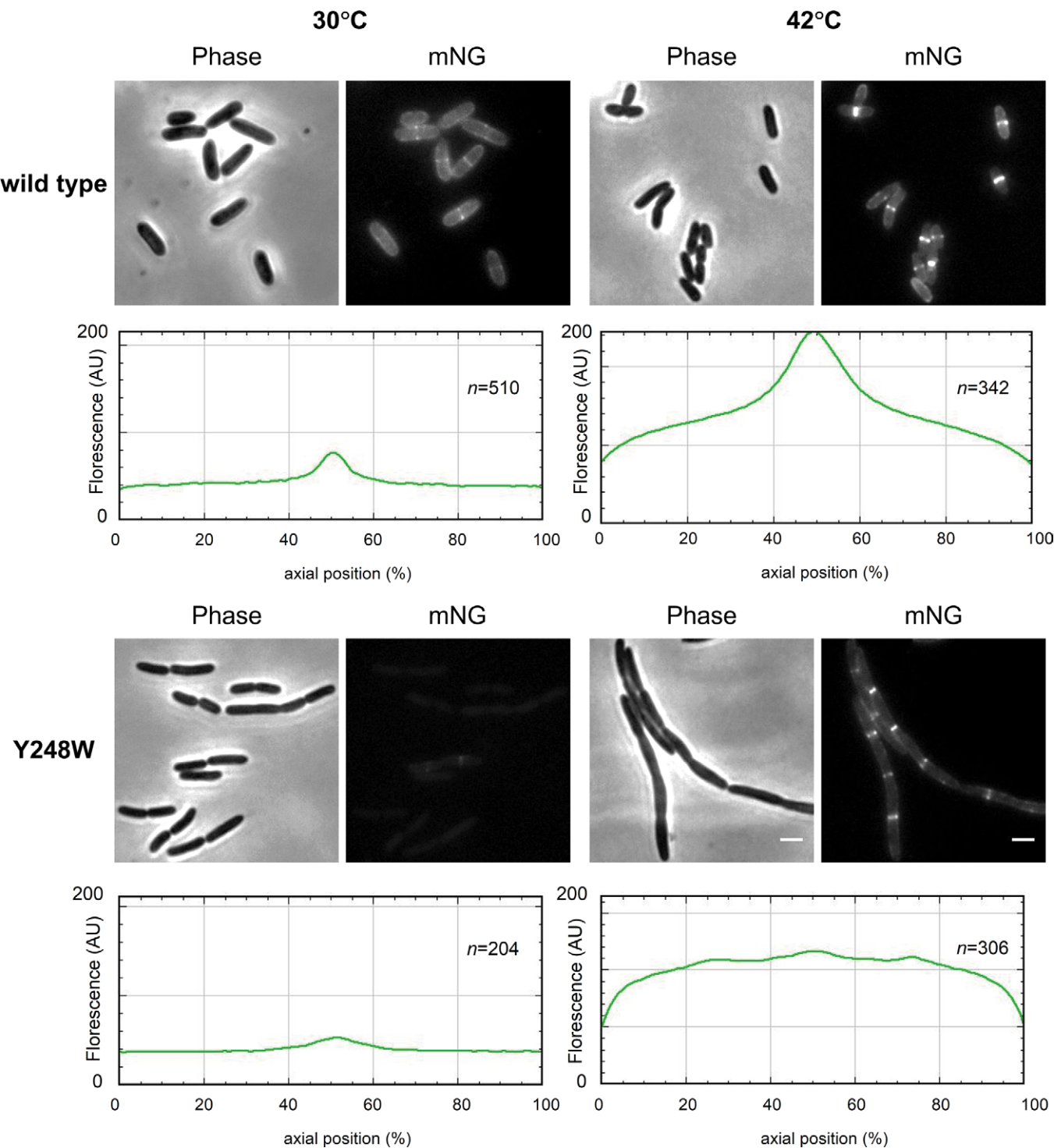

Supplement: FIG S4 [file mbo004184054sf4.pdf]

Supplementary Figure S5.

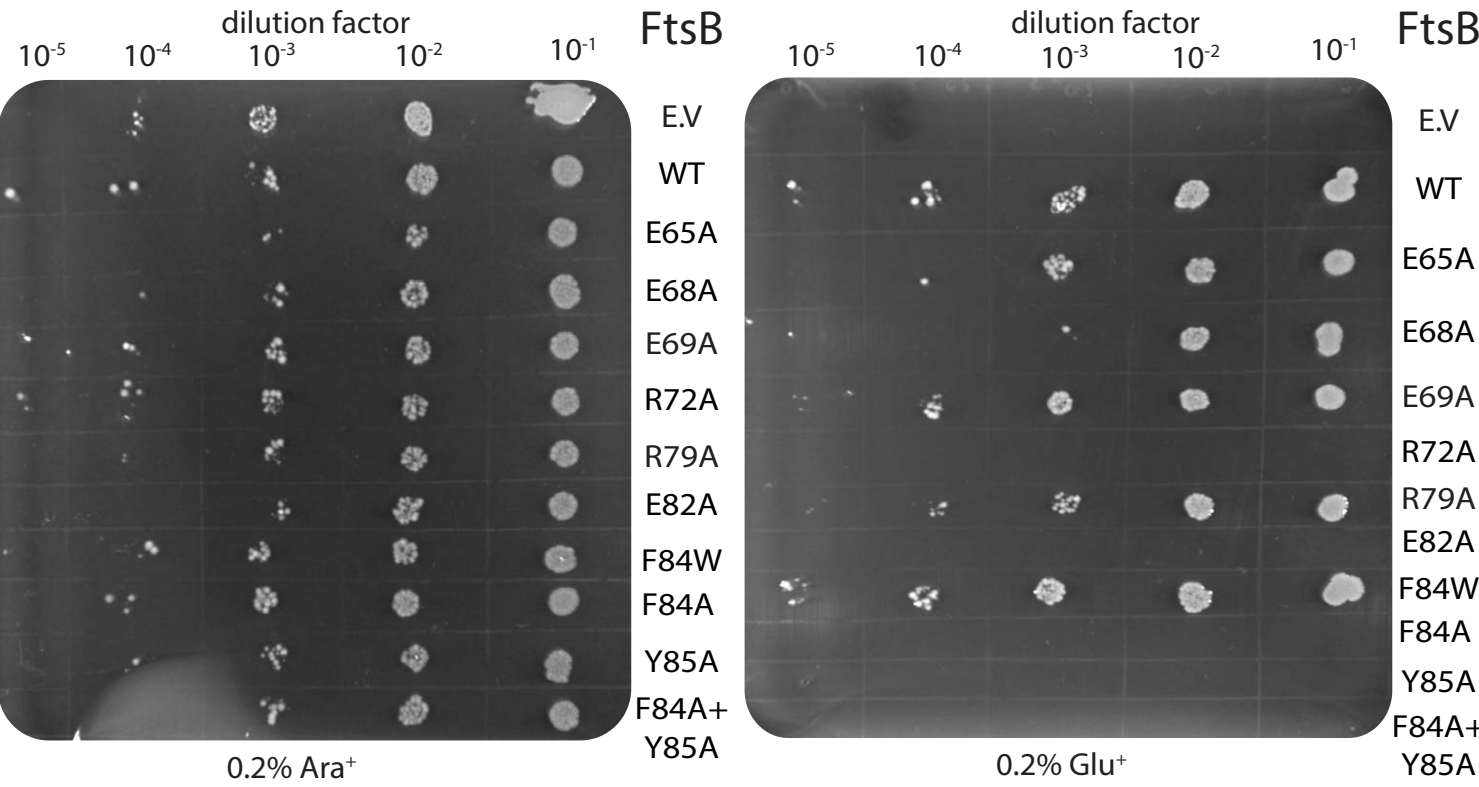

Supplement: FIG S5 [file mbo004184054sf5.pdf]
