# Supplementary material for: Structural Analysis of the Interaction between the Bacterial Cell Division Proteins FtsQ and FtsB
Source: mBio. 2018 Sep 11;9(5):e01346-18. doi: 10.1128/mBio.01346-18 (PMC6134095; doi:10.1128/mBio.01346-18)

Supplementary Figure S2.

N-terminus	formula, sequence	mw [g*mol^{-1}]	calculated m/z	found m/z
**Ac**	$C_{126}H_{203}N_{39}O_{41}$, QEALEERARNELS(Nle)TRPG ETFYRL-NH_2	2919.5	1460.25 / 973.83 730.63 / 584.7	1461.0 / 974.3 / 731.0
**FITC PEG**	$C_{151}H_{223}N_{41}O_{48}$ QEALEERARNELS(Nle)TRPG ETFYRL-NH_2	3411.6	1706.3 / 1137.87 853.65 / 683.12	1707.2 / 1138.3 854.0 / 638.3

a)

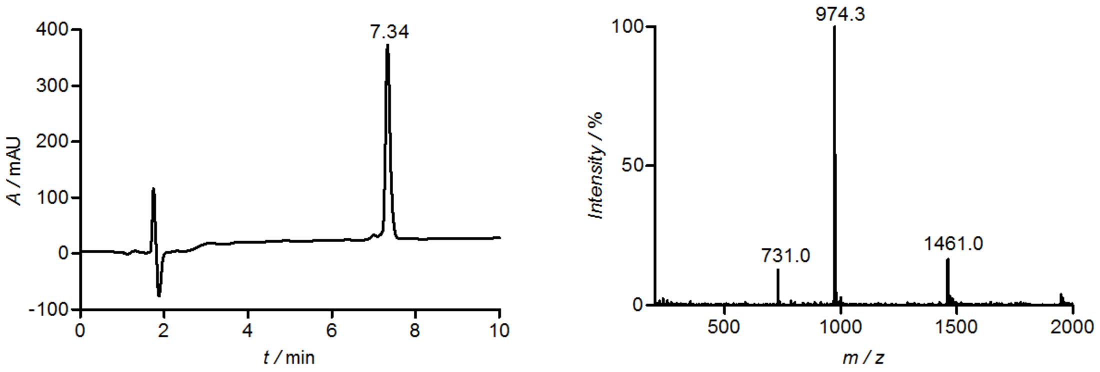

b)

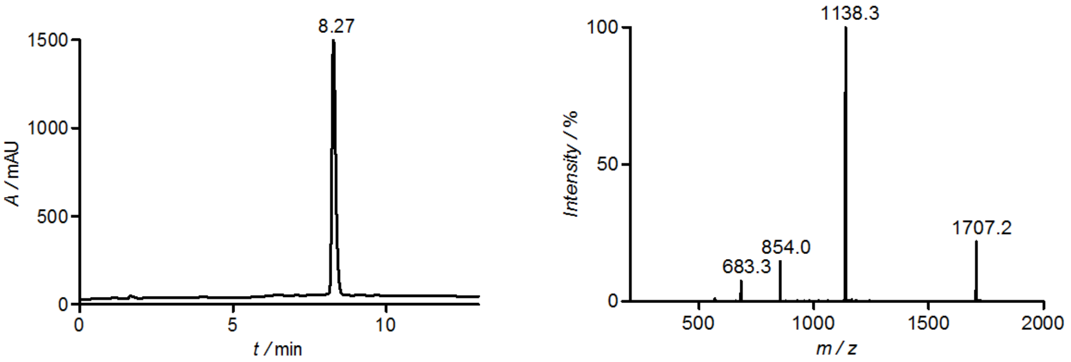

Supplement: FIG S2 [file mbo004184054sf2.pdf]
