# Supplementary material for: Structural Analysis of the Interaction between the Bacterial Cell Division Proteins FtsQ and FtsB
Source: mBio. 2018 Sep 11;9(5):e01346-18. doi: 10.1128/mBio.01346-18 (PMC6134095; doi:10.1128/mBio.01346-18)
Supplement: FIG S6 [file mbo004184054sf6.pdf]

Supplementary Figure S6.

A

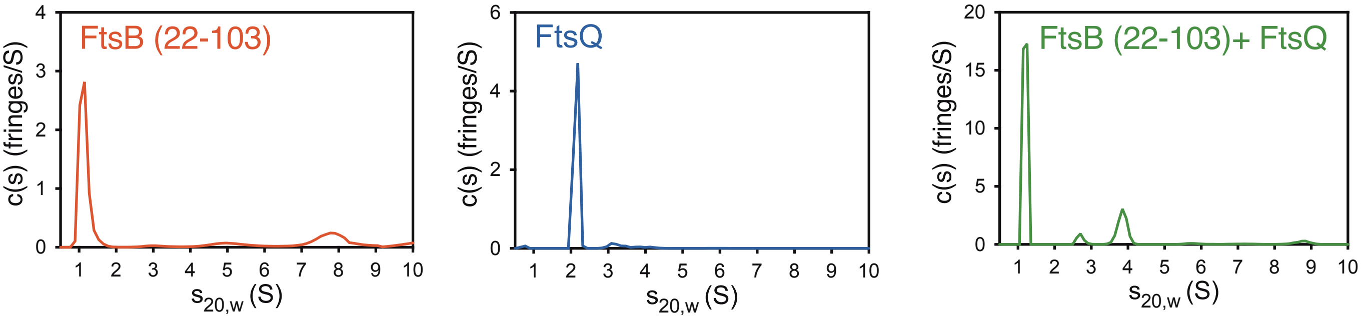

B

Protein	SEC-MALS Molar mass (kDa)	AUC Sedimentation coefficient ($S_{20,w}$)	AUC Calculated mass (kDa)
FtsB (22-103) 12.4 kDa	11.4	1.1	11.0
	65.5	3.5	58.6
	119	5.1	104
		8.0	203
FtsQ 26.2 kDa	27.9	2.2	22.9
		3.2	42.3
FtsB+FtsQ 38.6 kDa	21.9	1.2	12.0
		2.71	41
	67.8	3.84	69
